# Correction: *Trichlorobacter ammonificans*, a dedicated acetate-dependent ammonifier with a novel module for dissimilatory nitrate reduction to ammonia

**DOI:** 10.1093/ismejo/wrad018

**Published:** 2024-01-20

**Authors:** Dimitry Y Sorokin, Tamara V Tikhonova, Hanna Koch, Eveline M van den Berg, Renske S Hinderks, Martin Pabst, Natalia I Dergousova, Anastasia Y Solovevac, Gijs J Kuenen, Vladimir O Popov, Mark C M van Loosdrecht, Sebastian Lücker

Correction to: *The ISME Journal*https://doi.org/10.1038/s41396-023-01473-2, published 13 July 2023

There is a mistake in the culture collection number in the new species diagnosis on page 1647. Instead of UNIQEM 10005, it should read **UNIQEM U1005**. The corrected protologue is presented below.

**Description of *Trichlorobacter ammonificans* sp. nov**.

am.mo.ni’fi.cans. N.L. neut. n. *ammonium*, ammonium; L. ending -*ficans* (from L. v. *facio, facere*, to make); N.L. masc. part. adj. **a*mmonificans*, ammonifying.

Cells are comma-shaped, 1.5–4 x 0.5 μm, motile with a single polar flagellum, and tend to form semicircles and aggregates in old cultures. The culture has a distinctive pink colour, concentrated cell biomass is red. Colony formation was not observed. The dominant polar lipid fatty acids included unsaturated 16:1ω9c (differentiating the novel isolate from the closely related *Trichlorobacter* species) and two saturated fatty acids commonly present in the known *Trichlorobacter* species, 14:0 and 16:0 (Table S5). The organism is an obligate anaerobe with a very restricted metabolism limited to acetate-dependent dissimilatory nitrate/nitrite ammonification. H2 can be used as an additional (to acetate) electron donor. Weak growth was also observed with succinate, with only partial nitrate reduction to nitrite. The substrates tested negative with nitrate as acceptor included formate, ethylene glycol, glycine, ethanol, propionate, propanol, lactate, pyruvate, butyrate, butanol, malate, fumarate, valerate, yeast extract, and peptone. The electron acceptors tested negative with acetate as donor included different forms of Fe(III), AQDS, MnO_2_, arsenate and arsenite, selenate and selenite, fumarate, S_8_, and S_2_O_3_^2−^. The organism is an alkalitolerant neutrophile, growing optimally at pH 6.8–7.2 but tolerating pH values up to 8.5. The optimal growth temperature is 28–30°C. The genomic G + C content is 61.3%. The type strain is G1 (DSM 105480, UNIQEM U1005) isolated from activated sludge.

